# A case-control study on the driving factors of childhood brain volume loss: What pediatricians must explore

**DOI:** 10.1371/journal.pone.0276433

**Published:** 2022-12-30

**Authors:** Richard Sungura, Gabriel Shirima, John Spitsbergen, Emmanuel Mpolya, John-Mary Vianney

**Affiliations:** 1 Department of Health and Biomedical Sciences, School of Life Science, Nelson Mandela- African Institution of Science and Technology, Arusha, Tanzania; 2 Department of Neuroscience, Western Michigan University, Kalamazoo, MI, United States of America; Human Genetics and Genome Research Institute, National Research Centre, EGYPT

## Abstract

**Background:**

The brain volume loss also known as brain atrophy is increasingly observed among children in the course of performing neuroimaging using CT scan and MRI brains. While severe forms of brain volume loss are frequently associated with neurocognitive changes due to effects on thought processing speed, reasoning and memory of children that eventually alter their general personality, most clinicians embark themselves in managing the neurological manifestations of brain atrophy in childhood and less is known regarding the offending factors responsible for developing pre-senile brain atrophy. It was therefore the goal of this study to explore the factors that drive the occurrence of childhood brain volume under the guidance of brain CT scan quantitative evaluation.

**Methods:**

This study was a case-control study involving 168 subjects with brain atrophy who were compared with 168 age and gender matched control subjects with normal brains on CT scan under the age of 18 years. All the children with brain CT scan were subjected to an intense review of their birth and medical history including laboratory investigation reports.

**Results:**

Results showed significant and influential risk factors for brain atrophy in varying trends among children including age between 14-17(OR = 1.1), male gender (OR = 1.9), birth outside facility (OR = 0.99), immaturity (OR = 1.04), malnutrition (OR = 0.97), head trauma (OR = 1.02), maternal alcoholism (OR = 1.0), antiepileptic drugs & convulsive disorders (OR = 1.0), radiation injury (OR = 1.06), space occupying lesions and ICP (OR = 1.01) and birth injury/asphyxia (OR = 1.02).

**Conclusions:**

Pathological reduction of brain volume in childhood exhibits a steady trend with the increase in pediatric age, with space occupying lesions & intracranial pressure being the most profound causes of brain atrophy.

## 1. Introduction

The human brain is an essential component of the central nervous system and handles many functions, including neurocognitive aspect of brain functions especially under the control of frontal and temporal lobes [1]. The functions of the brain are the results of the development of the neuro-anatomic organ whose development begins in the 16th week by neuroblasts multiplication [2], and shows an orderly pattern of adult growth [3]. Although the formation of the human brain begins early following conception, it goes through a time of normal childhood development [3] and involutes in elderly people with a progressive volume loss demonstrated by an increase in sulcal spaces.

The atrophy of the senile brain is known to occur from the 50s and beyond following the plateau brain development stage that occurs after 40 years [4]. The age and mass of the body (BMI) are both recognized for the reduction in brain volume [5]. When this pattern is taken in chronology, it is unusual to occur in childhood or at around 40 years’ period since it is the plateau stage of human brain development [6]. Therefore, when premature atrophy emerges, it is considered to be pathological and therefore, intensive search for an offending source is deemed essential.

A prominent clinical indication of the disordered brain is the change in mental status. These modifications include several functions such as speed, reasoning, memory, problem-solving, reading ability, learning ability, attentiveness, mood and an individual’s overall personality [7]. These manifestations are commonly encountered in individuals with brain volume loss. This clue adds more gravity on the need to seek for a deeper knowledge on the driving factors responsible for childhood brain atrophy in various places.

Prematurity, central nervous system infections such as meningitis, HIV encephalopathy [8], and cerebral malaria have all been identified as causes of brain shrinkage in different parts of the brains [8]. Head trauma is also an important concern, particularly in the context of consciousness loss [9], metabolic conditions such as Cushing syndrome [10], drug-related causes such as anti-epileptics, chemotherapy for cancers [11], maternal alcoholism, particularly more than 300ml/day during pregnancy [12], seizure disorders, particularly status epilepticus [13], radiation-induced brain injury [14], perinatal injury [15], perinatal disorders [16]. Other factors include malnutrition, both in terms of avitaminosis-B and protein energy malnutrition; however, in the continuous examination of non-vegetarian populations, vitamins B1 and B12 are known to be too dynamic, with unstable levels in the blood [17]. The determinants of brain atrophy in children may thus be affected by the population’s geography and socio-culturalenvironment.

This study therefore examined and quantified the influence of different predictor variables for brain atrophy in children under the age of 18 in northern Tanzania.

This study was very important and relevant in the planning and designing of brain atrophy mitigation plans in Tanzania and adjacent nations whose incidence in the northern regions currently stands at 14.06 percent [18]. The outcomes of this study are crucial for course- specific brain atrophy intervention approaches.

## 2. Materials and methods

### 2.1 Study area and design

Four facilities were included in the study, namely Agakhan Health Center, the Arusha Lutheran medical Center, Afyamax polyclinic all in Arusha region and the Kilimanjaro Christian Medical Center in Kilimanjaro region of Tanzania. The centers were picked intentionally for individuals residing inside Northern Tanzania on the basis of availability and access to CT scan imaging services. During this study period, the two locations, namely Tanga and Manyara, did not have functioning CT scans. Their patients were however sampled by referral system set by the national health care network system.

### Sample size

Charan & Biswas, 2013, derived the sample size from a statistical formula, by the guide that part of the investigation concerned quantitative variables in case control [19].


$$N=\frac{r+1}{r}\frac{SD^{2}(Z_{\beta }+Z_{1‐\alpha /2})2}{d^{2}}$$

Where as:

Standard variable Z1-α /2 standard (the error is 1.96 at 5% type 1 (P<0.05). The standard deviation value was derived from Holger Schmidt’s previous work and 2.3 was found [20].

d = The expected mean difference between case and control but in this case, half the value of the SD was considered to be 1.15.

*r* = Ratio of control to cases, 1 for equal number of Case and Control.

Z = Standard normal power variate; the value is 1.28 for 90% power.

In this study, the 90% power was selected with SD (2.3) and d values (1.15), the sample size was calculated to be 168 and when the ratio of 1:1 [21] was considered the study then recruited 168 cases and 168 controls.

### 2.2 Inclusion and exclusion criteria

Cases and controls were included after meeting inclusion criteria by being (i) less than 18 years of age; (ii) have performed brain CT scan. (iii) Accessibility and availability of quality CT scan image of patients. (iv) Presence of the biological mother for the birth and early infancy history.

The exclusion criteria of the study involved (i) Individuals of 18 years and above. Children who were not born and raised in Northern Tanzania were excluded.

### 2.3 Matching method

The matching criteria involved age and sex. Case-control ratio of 1:1 with age interval of 2 years was considered.

### 2.4 Procedures

A radiologist examined the Brain CT-scan images, which evaluated sulcal width, lateral ventricular body width and the Evans index using the three familiar radiological linear procedures. Diagonal multi-linear (DBF) approach was calculated to identify the presence or absence of brain atrophy for those who qualified for the use of this technique [22].

**Fig 1:**
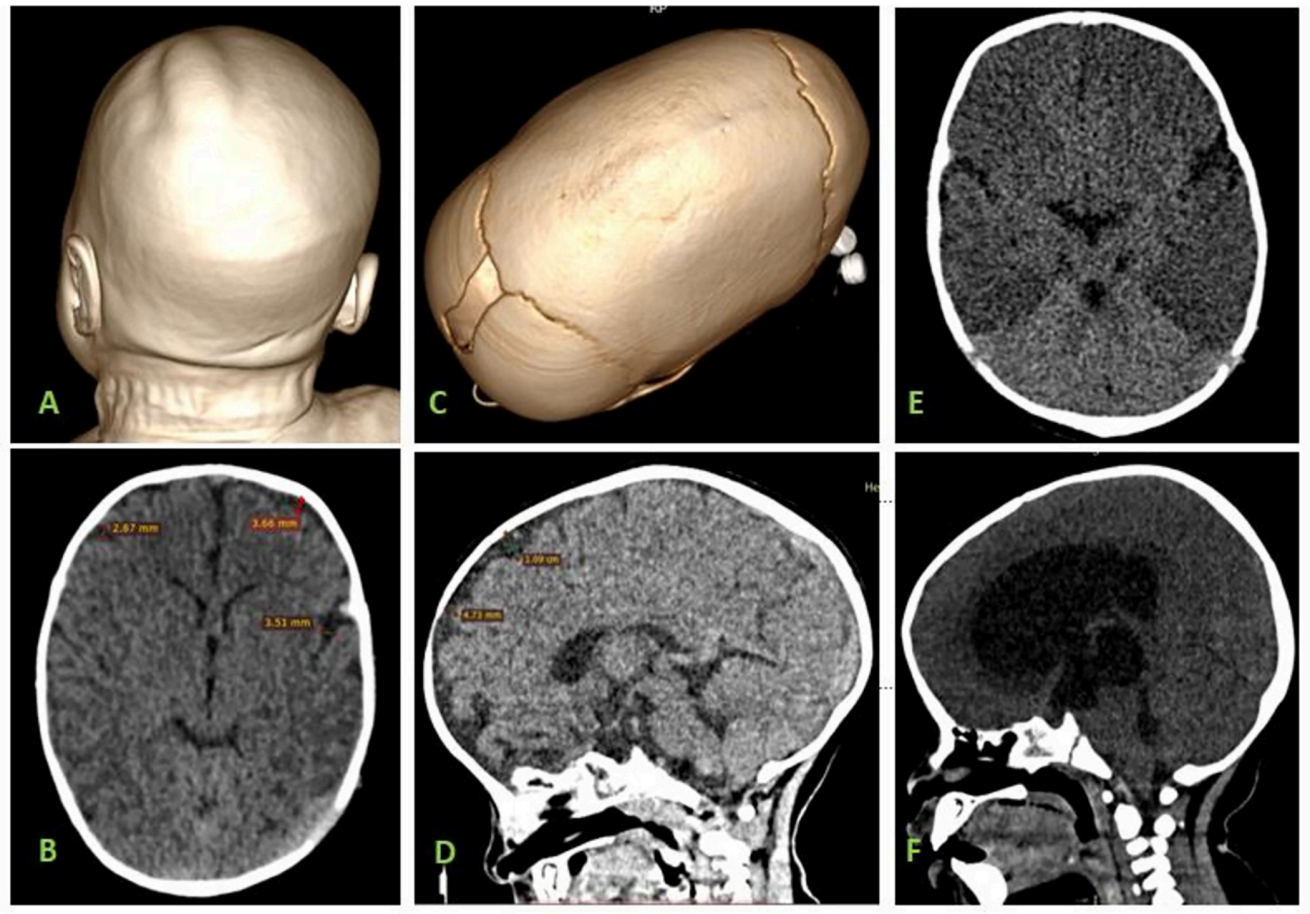
Selected head CT scans showing varying causes of brain volume loss. A: Volume rendering 3D CT scan image of a 3 months’ child with birth related head injury presenting with cephalohematoma at the vertex. B: Axial CT scan image of the same child A showing diffuse reduction in brain volume in cortical regions as evidenced by prominent sulci. C: Volume rendering 3D image of 1-year male child showing premature closure of the sagittal suture with overall increase in anterior-posterior diameter of the head(craniosynostosis). D: Sagittal CT brain image of the same child C showing loss of brain volume in the frontal lobes showing gross deviation from the calvarium with prominent CSF spaces. E: A 2 months’ female child presenting with diffuse reduction in cerebral hemispheric density sparing the cerebellum (White cerebella sign) representing hypoxic ischemic encephalopathy with early bi-temporal cerebral atrophy. F: A 4 months’ male child showing increased intracranial pressure evidenced by effacement of sulci and gyri as a result of ballooning of the lateral and 3^**rd**^ ventricles. The 4^**th**^ ventricle is small due to obstructive hydrocephalus following aquiductal stenosis.

The collection of data included questionnaires designed to assess the presence of 10 non- demographic categories of risk factors using primary information from mothers of children covering medical history from antenatal to adolescents, examination of obvious risk factors through image analysis such as tumors, intracranial pressure, hypoxic-ischemic encephalopathy as well as head injury was conducted (Fig 1). Additional data as age and sex were considered from hospitals data base. After parents’ approval, children whose results for HIV tests were not found were subjected to such tests.

### 2.5 Statistical analysis

The descriptive statistics such as frequency, percentage and graphical visualization (bar graphs) were produced. Some inferential statistics were also performed. A simple linear regression model was produced to assess the impact of risk factors such as head trauma, birth asphyxia among others toward the response variable (brain atrophy). The significant risk factors were then exported to the multiple linear regression analysis. All liner regression assumptions were checked including normality of the continuous variables. Shapiro-Wilk test and variance inflation factor (VIF) were used to check for normality and collinearity tests respectively. Variables with VIF above 10 were removed from the model otherwise they were retained in the model development. All tests were performed at 5% confidence level. Also, the coefficient of determination (R-square) was used to check for model goodness of fit. The R-statistical software version 4.0.1 was used for all analyses.

### 2.6 Ethical consideration

For ethical approval of this study, the Northern Zone Health Research Ethics Committee (KNCHREC) was consulted, and a research permit number KNCHREC 0010 dated 20^th^ December 2019 was obtained. Covering letters were then sent to each health facility, requesting permission to collect and use data from their archives within the parameters of this study at no additional cost. Once the study was explained to the participants, their parents or guardians freely consented to their participation by signing pre-written forms. Patients’ names and other personal identifiers were not recorded in order to maintain confidentiality. The retrospective data components were anonymized.

## 3. Results

### 3.1 Age demographic characteristics of the participants

**Fig 2:**
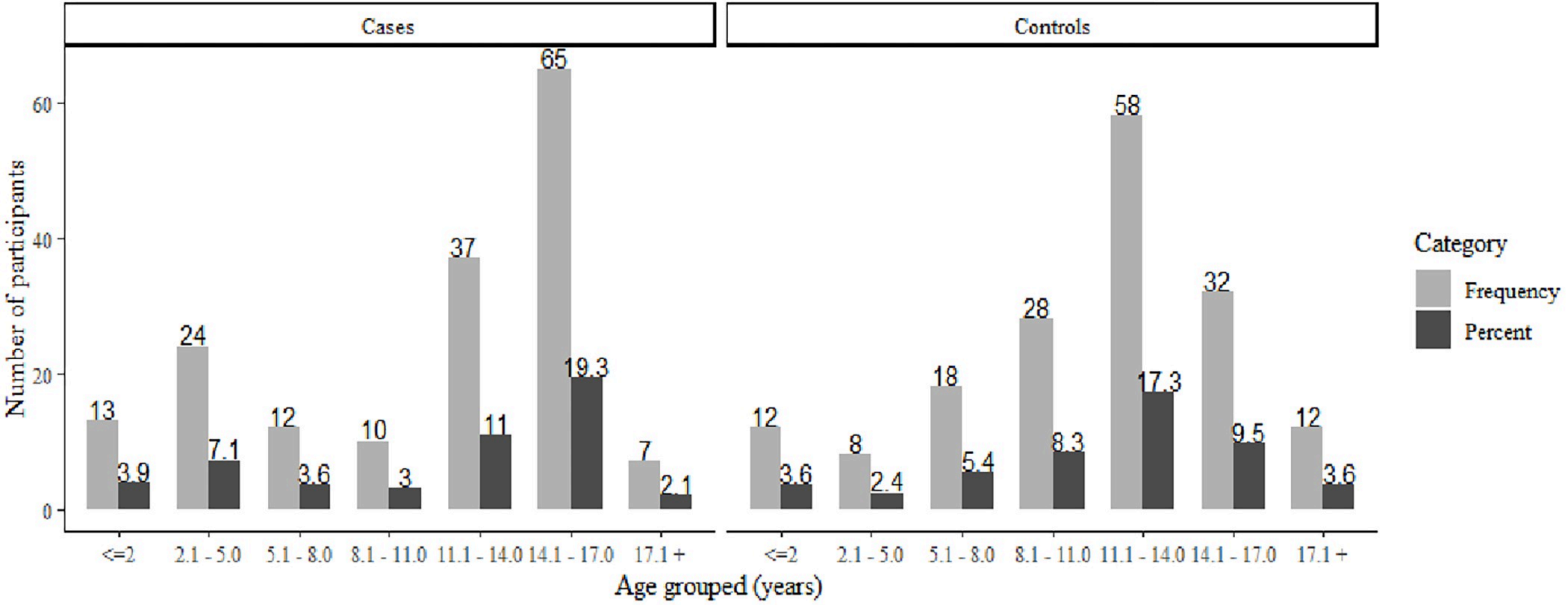
Age demographic distribution of the participants. Children from both groups of cases and controls of the brain atrophy are skewed more toward the right extreme of age with the peak age between 14.1–17.0 years.

Our results presented varying age of subjects in the study in which the peak ages of participants were noted at the age between 14.1–17.0 years among cases of brain atrophy as well as control groups. The second peak was noted at the age of 11.1–14.0 years among cases and the age between 14.0–17.0 years among control group showing an overall distribution of participants being skewed toward the age of 14.1–17.0 in both cases and controls of this study (Fig 2).

### 3.2 Gender demographic characteristics of the participants

**Fig 3:**
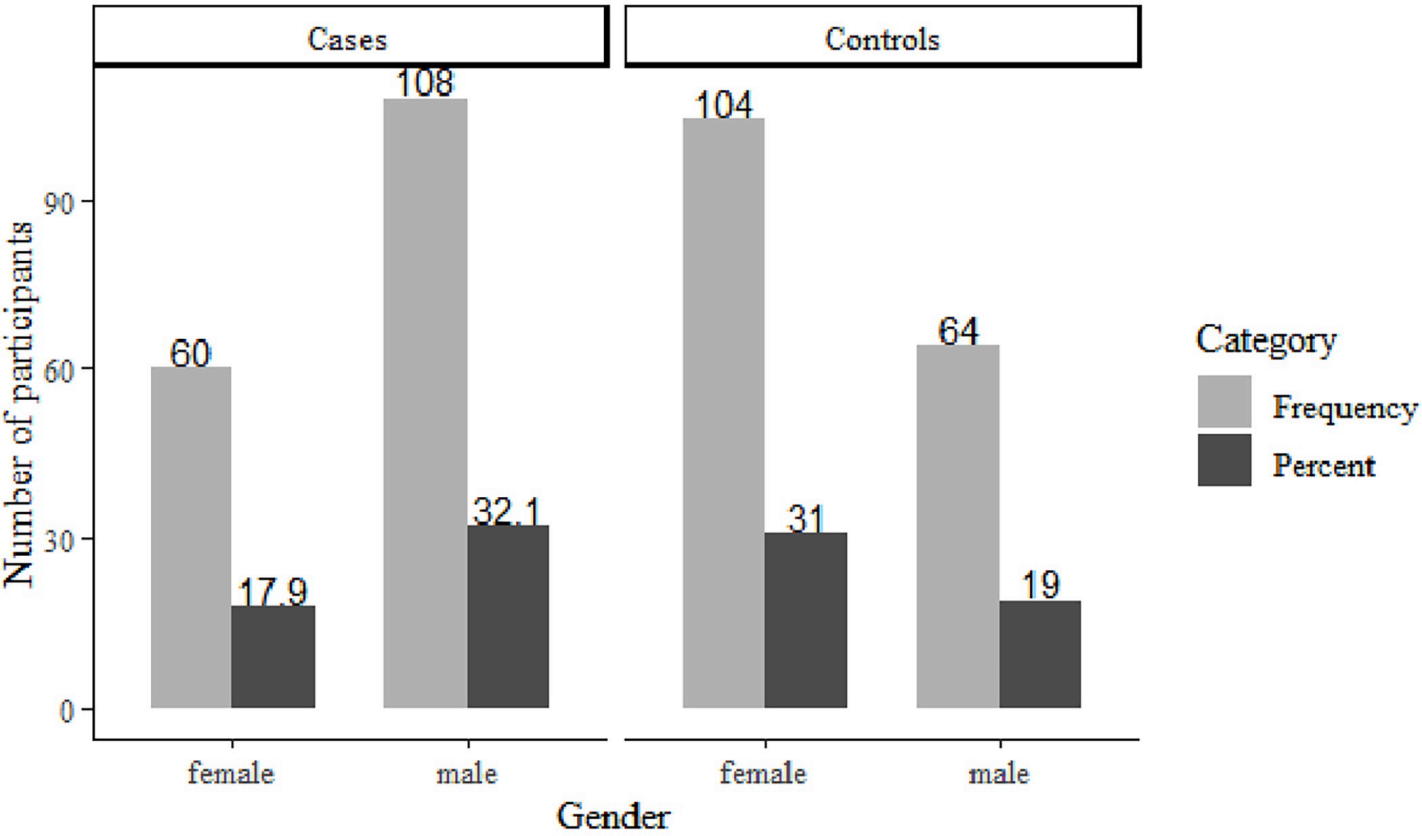
Demographic picture of gender distribution of participants. A higher proportion of childhood brain atrophy is shown in male children of the sampled population than in female children.

The results of this study show that male children dominated the population with brain atrophy in which a total of 64.2% of the cases were male children while 35.8% of this small population was female children. Paradoxically, the gender distribution of the sampled population of the control was different in which female children were the majority in this small group accounting for 104 subjects out of 168 representing 61.9% of the children with normal brain while male children were only 64 representing 38.1% of the control group. In totality the study was dominated by atrophied brain from the male children accounting for 32.1% of the whole population (Fig 3).

### 3.3 The driving predictors of childhood brain atrophy in northern Tanzania

Varying risks factors for the prediction of brain atrophy occurrence which were considered returned different results involving; age, gender and other determinants of brain atrophy.

Among the demographic related predictive factors for the occurrence of brain atrophy; age showed significant contribution at the age categories of 14.1–17.0 years with OR = 1.1 among cases and OR = 1.0 among controls. Similarly, at the age above 17.1 years the OR = 1.1 was seen among cases and OR = 0.96 among control group. When age was considered as a continuous variable results showed statistical significance in both cases and controls in which the (OR = 1.01, p-value = 0.03) was found among cases while (OR = 0.99, p-value = 0.0349) was the observation in the control group.

Gender category showed significance in male children with OR = 1.1 among cases of brain atrophy while this was not significant among control group with p-value of 0.0899 despite OR = 1.0.

The other non-demographic risk factors which were tested involved birth outside facility (birth before arrival), immaturity, central nervous system infection, malnutrition, head trauma, maternal alcoholism, convulsive disorders with antiepileptic drugs, radiation injury, intracranial pressure and space occupying lesion, and birth injury. Among these risk factors the univariate analysis showed statistic significant factors involving; central nervous system infection (meningitis or cerebral malaria) with (OR = 1.0, p-value = 0.0137) among the cases and (OR = 0.99, p-value = 0.0037) among the controls. Trauma showed (OR = 1.02, p-value = 0.06050 among cases of brain atrophy. Convulsive disorders & antiepileptic drugs showed (OR = 1.0, p-value = 0.0006) among cases of brain atrophy and (OR = 0.97, p-value = 0.0450) among the controls. Intracranial pressure and space occupying lesions returned (OR = 1.01, p-value 0.00002) among the cases of brain atrophy. Birth injury (birth asphyxia) showed (OR = 1.02, p-value = 0.0152) among cases of brain atrophy and (OR = 1.0, p-value = 0.0005) among controls (Table 1).

**Table 1:** Factors driving the occurrence childhood brain atrophy in Northern Tanzania. Simple logistic regression analysis showing demographic and non-demographic factors responsible for childhood brain atrophy. Most of non-demographic factors including CNS infection, trauma, convulsions and drugs, SOL&ICP and birth injury are influential independent variables. Upper extreme of age and male gender are significant among demographic factors.

	CASES					CONTROLS				
			95% CI					95% CI		
Variables	N (%)	OR	Lower	Upper	p-value	N (%)	OR	Lower	Upper	P-value
**Age**										
≤ 2	13(7.7)	0.97	-0.068	0.0100	0.1441	12(7.1)	1.0	-0.02	0.04	0.4333
2.1–5.0	17(14.3)	1.0	-0.043	0.076	0.5790	8(4.8)	1.0	-0.03	0.04	0.6238
5.1–8.0	15(7.1)	0.98	-0.07	0.047	0.6554	18(10.7)	1.02	-0.001	0.039	0.0637
8.1–11.0	13(6.0)	1.0	-0.062	0.065	0.9618	28(16.7)		-0.0745	0.0003	0.0709.
11.1–14.0	39(22.0)	1.1	-0.00	0.103	0.0507.	58(34.5)	1.0	-0.011	0.019	0.2748
14.1–17.0	66(38.7)	1.1	0.008	0.106	0.0220 *	32(19.0)	1.0	-0.021	0.011	0.9147
17.1+	5(4.2)	1.1	0.021	0.191	0.0146 *	12(7.1)	0.96	-0.073	-0.011	0.00879 ***
**Gender**										
Male	99(64.3)	1.92	0.005	0.057	0.0217*	104 (61.9)	1.0	-0.001	0.020	0.0899.
Female	69(35.7)	0.99	-0.041	0.003	0.3825	64(38.1)	0.99	-0.022	0.007	0.0899.
Birth-Outside (YES)	22(13.1)	0.99	-0.042	0.038	0.93	8(4.8)	0.97	-0.066	0.007	0.0005***
Birth-Outside (NO)	146(86.9)	1.0	-0.042	0.038	0.93	160(95.2)	1.0	-0.066	0.007	0.0005***
Immaturity (YES)	25(14.9)	1.04	-0.003	0.080	0.0685.	76(45.2)	1.0	-0.012	0.022	0.1580
Immaturity (NO)	143(85.1)	0.98	-0.003	0.080	0.0685.	92(54.8)	0.99	-0.002	0.012	0.1580
CNS_infection (YES)	56(33.3)	1.0	0.006	0.052	0.0137*	24(14.3)	0.99	-0.034	0.013	0.0037**
CNS_infection (NO	112(66.7)	0.99	-0.002	0.043	0.0137*	144(85.7)	1.0	-0.034	0.013	0.0037**
Malnutrition (YES)	12(7.1)	0.97	-0.079	0.022	0.2700	12(7.1)	0.98	-0.049	0.0169	0.0618.
Malnutrition (NO)	156(92.9)					156(92.9)	1.02	0.049	0.0179	0.0618.
Trauma (YES)	46(27.4)	1.02	-0.029	0.0605	0.0137*	26(15.5)	0.97	-0.029	-0.00605	0.5710
Trauma (NO)	122(72.6)	0.98	-0.029	0.0605	0.0137*	142(84.5)	1.00	-0.029	-0.00605	0.5710
Metabolic (YES)	15(8.9)	1.0	-0.089	0.0004	0.0522.	6(3.6)	0.98	-0.055	0.030	0.4940
Metabolic (NO)	153(91.1)	0.99	-0.089	0.0004	0.0522.	162(96.4)	1.00	-0.055	0.030	0.4940
Drug related (YES)	28(16.7)	1.00	-0.072	0.020	0.0006***	8(4.8)	0.97	-0.022	0.014	0.0450*
Drug related (NO)	140(83.3)	0.99	-0.072	0.020	0.0006***	160(95.2))	1.00	-0.022	0.014	0.0450*
Radiation injury (YES)	2(1.2)	1.06	-0.11	0.23	0.497	2(1.2)	0.96	-0.036	-0.0108	0.1740
Radiation injury (NO)	166(98.8)	0.98	-0.11	0.23	0.497	166(98.8)	1.00	-0.036	-0.0108	0.1740meta
SOL_ICP (YES)	23(13.7)	1.01	-0.11	0.04	0.00002***	4(2.4)	0.99	-0.048	0.0369	0.8720
SOL_ICP (NO)	145(13.7)	0.99	-0.11	0.04	0.00002***	164(97.6)	1.00	-0.048	0.0369	0.8720
Birth injury (YES)	21(12.5)	1.02	0.004	0.038	0.0152*	8(4.8)	0.99	-0.035	0.032	0.0005***
Birth injury (NO)	147(87.5)	0.99	-0.004	0.038	0.0152*	160(95.2)	1.00	-0.035	0.032	0.0005***
Age (Continuous)	168(100.0)	1.01	0.003	0.0076	0.00006***	168(100.0)	0.99	-0.002	-0.0008	0.0349 **

Simple linear regression was used to compute the relationship that exists between cases and factors that determines the occurrences of DBF, similarly, the same approach is used for controls too. The computed estimates were exponentiated to produce odds ratio for each factor. The 5% confidence level used. Significant codes: p<0.1

*p<0.05

**p<0.01 and ***p<0.001. The sample size of cases is 168 and controls is 84 individuals.

### 3.4 The most influential predictive factors of childhood brain volume loss

The following are the most influential factors in childhood brain volume loss returned from the multivariate analysis of the study after the significant categories in the univariate analysis which were exported into a multivariate analysis, these driving factors showed statistical significance; The convulsive disorders & antiepileptic drugs had (OR = 1.02, p-value = 0.0089), intracranial pressure and space occupying lesion returned (OR = 1.03, p-value = 0.0005), birth injury (birth asphyxia) showed (OR = 1.01, 0.02932) among cases of brain atrophy but also (OR = 0.90, p-value <0.001) among the controls. Head trauma showed (OR = 1.02, p-value = 0.00461), Central nervous system infection returned (OR = 1.02, p-value = 0.00113) among cases of brain atrophy and also (OR = 0.97, p-value = 0.0010) among the control subjects. Finally, the model showed a relative risk of 0.516 with p-value of <0.0001 among cases of brain atrophy while 0.496 with p-value of 0.0001 was noted among the controls (Table 2).

**Table 2:** The most influential drivers of brain atrophy among children. The multiple logistic regression analysis show none of the demographic factor as a significant variable in causing brain atrophy. The order of influence and significance is represented by space occupying lesions, central nervous system infection, convulsions and anti-epileptic drugs, and birth injury.

	CASES				CONTROLS			
		95% CI				95% CI		
Variables	OR	Lower	Upper	p-value	OR	Lower	Upper	P-value
**Gender**								
Male	1.02	-0.008	0.042	0.1822	1.01	-0.007	0.0269	0.0159*
Female	0.99	-0.021	0.004	0.2825	0.96	-0.013	0.022	0.1622
Immaturity (YES)	1.01	-0.032	0.044	0.757	0.99	-0.013	0.008	0.6065
Metabolic (YES)	1.02	-0.003	0.038	0.0858.	0.98	-0.045	0.007	0.0541.
Drug related (YES)	1.02	0.004	0.031	0.0089**	0.96	-0.006	0.004	0.1614
SOL_ICP (YES)	1.03	0.012	0.044	0.0005***	1.01	-0.039	0.053	0.7702
Age (Continuous)	1.00	0.003	0.007	0.0002***	0.99	-0.002	0.004	0.2443
Birth injury (YES)	1.01	0.001	0.031	0.02932 *	0.90	-0.147	-0.055	< 0.001***
Trauma	1.02	0.005	0.026	0.00461 **	1.00	-0.003	0.030	0.1026
CNS infection	1.02	0.016	0.007	0.00113 **	0.97	-0.055	-0.0015	0.0010**
**R** ^**2**^	**0.516**			**<0.0001** ***	**0.496**			**0.0001** ***

Finally, a multiple linear regression was used to compute the relationship that exists between cases and factors that determines the occurrences of DBF, similarly, the same approach is used for controls too. The computed estimates were exponentiated to produce odds ratio for each factor. The 5% confidence level used. Significant codes

p<0.1

*p<0.05

**p<0.01 and ***p<0.001

## 4. Discussion

Even though the study involved all age groups less than 18 years, there was a varying trend of participants in this hospital based study. The most dominant age group of children who participated in this study was centered at the age between 14.1–17.0 years in both cases and controls. This may suggest that the given age range is the most active age of adolescence more susceptible to environmental risk factors that drive them to the need of medical attention especially the neuroimaging related to increased incidences of trauma. The study by Meulepas et al., found an increasing need for medical imaging among children over the age of ten in the Netherlands, which is similar to the findings of this study [23]. Furthermore, because international standards and regulations advocate against subjecting young children to unnecessary imaging with ionizing radiation tools, it is likely that younger children who required medical images were subjected to MRI and trans-cranial ultrasound, particularly those under the age of two [24]. This is because the fontanels close at an estimated age of 18 months, making pediatric brain visualization via ultrasonography difficult after this age [25].

Surprisingly, while the whole study was generally dominated by male children with atrophied brain constituting 32.1% of all the participants, the same male children were the majority by 64.2% of all children in the sub-group of atrophied cases and only 35.8% of this sub-group were female children suggesting a very strong tendency of brain atrophy prevailing in the male gender. The results paradoxically showed a protective tendency among female children toward brain atrophy development as 61.9% of the control group was dominated by the female children and only 38.1% of this sub-group was represented by male children. Since male children have been associated with behavioral related risk factors linked to events of head trauma [26], they were subjected more in developing brain atrophy than female children. The study in Maasai community suggested that female children stay more in homes than male children and hence this could be the reason for less exposure to environmental risk factors [27]. Since most of the participants in this study were sampled in the age between 14.1–17 years, this may suggest that the activities of gonadal hormones (testosterone and estrogen) had taken place for male and female children’s secondary physical characteristics differentiation [28]. There is likelihood that estrogen is more protective and contributes to higher resilience against neuronal degeneration among female children. A study by DeCarli et al, 2005 suggested estrogen withdrawal was a reason for accelerated senile brain atrophy among female patients [29]. Another study also showed that low dose estrogen has neuronal protection against degeneration after spinal injury as per study by Samantaray et al, 2011 [30].

Varying risks factors for the prediction of brain atrophy occurrence which were considered returned different results on age, gender and other determinants thought to trigger and initiate brain volume loss in children. The factors which were considered in this study could be grouped as demographic and non-demographic factors. In these broad groups, age category showed significant influence among the demographic factors and that, this was found to be significant at the age between 14.1–17.0 years as well as above 17.0 years. When age was alternatively considered as a continuous variable it showed statistical significance in the univariate analysis.

This observation is accounted by the fact that as brain grows, there is increase in CSF spaces between the brain and the calvarium [31]. There is also more myelination of the brain with progressive reduction in water contents of the brain resulting in reduced plasticity of the brain mantle hence assuming more of its solid nature away from the calvarial intimacy [32]. This together with events of dehydration may lead to reduction in brain volume with the increase in age [33]. This is not considered to be a degenerative brain atrophy of the senile age.

When gender demographics were considered, the study returned statistical significance among male children in cases of brain atrophy only and this was not significant among controls. This finding suggests the existence of the resilient nature of the female brains against loss of brain volume in childhood [34].

Among the non-demographic risk factors which were studied involving immaturity, central nervous system infection, malnutrition, head trauma, maternal alcoholism, convulsive disorders with antiepileptic drugs, radiation injury, intracranial pressure and space occupying lesion and birth injury; only six risk factors returned statistical significant influence for developing brain atrophy in univariate analysis. These factors involved central nervous system infection, head trauma, convulsive disorders and antiepileptic drugs, intracranial pressure and space occupying lesions, and lastly birth asphyxia.

According to the study by Makene et al, incidences of premature deliveries (immaturity) and related morbidity have been reduced due to increased awareness and provision of neonatal care for mother and child [35], there is likelihood that such children do not suffer much hypoxia that may result in brain damage.

There is also overall reduction of malnutrition rate in Northern Tanzania despite few children from Maasai community due to migration tendency and nutritious food insecurity during dry seasons when cows are taken far for pastures depriving children from milk and meat supply [36].

Another socially significant parameter considered in this study was maternal alcoholism. While the condition has been linked to an increased risk of microcephaly [37], brain atrophy is a slightly different entity because it only considers the reduction in brain volume associated with calvarial intimacy loss [38]. This was only a trend discovered in this study, but not statistically significant in influencing brain atrophy causation. The finding can be attributed to different metabolic rates among pregnant mothers, with most of them having more efficient first pass metabolism, as well as genetic polymorphisms that exist in degrading alcohol to tolerance levels at the utero-placental exchange [39].

Generally malignant tumors with exception to lymphomas are rare in children [40]. The study encountered a negligible number of children with cancers of head and neck that mandated the need for radiotherapy of the head and neck region. Although radiotherapy is known to damage cells [41], its influence in this community was not statistically significant.

An in depth review of the six significant risk factors which were found in the multi variate analysis performed in this study, such factors can be further discussed in descending order of their influences based on odds ratios and p-values including; space occupying lesions and intracranial pressure, central nervous system infections, head trauma, convulsions and antiepileptic drugs, birth injury (birth asphyxia) and finally a continuous increase in age.

The space occupying lesions included lesions such as intracranial tumors, hydrocephalus and craniosynostosis [42]. In this study, this category was the most influential likely due to more than one mechanism of actions in developing brain atrophy. Studies have shown that these lesions result in brain volume loss by virtual of pressure effect or nutritional competition with the native brain cells [43].

The infections of the central nervous system are diverse but the most important in this study comes from episodes of cerebral malaria and meningitis in childhood [44]. Complicated infection associated with brain abscesses or tuberculoma had both inflammatory effect on brain cells and space occupying effects leading into loss of volume [45].

Trauma is very common risk factor especially to old children who are more active. Studies have shown that brain tend to loose volume when it has undergone contusion change especially when associated with loss of consciousness [46]. Furthermore, other authors have observed that the timing of brain atrophy ranges from three and eleven months after the incident of head trauma [42, 47]. It has also been reported that immediately after brain trauma, the injured tissue may provoke activation of macrophages which tend to migrate and consume the damaged cells and in doing so they may go further engulfing the innocent brain cells and tissues leading to a far reaching brain volume loss [48].

Convulsions in childhood may come as result of many reasons but not limited to genetic epileptic syndromes such as Lennox-Gastaut syndrome [49], West syndrome [50] and childhood absence epilepsy [45]. Convulsive disorders are rooted from explosive or abnormal electrical discharges generated from the neuronal cells of the brain. They may also occur after brain damage through trauma, mesial temporal lobe sclerosis [51] or even abnormal neuronal migrational anomalies such as heterotopias [52]. These anatomical changes may result in convulsions associated with jerky body movements, behavioral arrest and sometimes involuntary activities such as urine incontinence and tongue biting. In this study, children with convulsive disorders presented with loss of brain volume in a significant number. It is reported that epileptic discharges result in high usage of energy in the form of ATP resulting in tissue necrosis due to local energy depletion and in turn this leads to loss of brain volume [53]. It is also said that antiepileptic drugs are associated with brain atrophy causation [54]. While in a general view, the goal of anti-epileptic drugs such as carbamazepine is to control seizures which eventually may lead to focal or generalized atrophy of brain, the confounding effects of convulsive disorders and anti-epileptic drugs toward causation of brain atrophy is vaguely separated in the body of literature and hence this was a rationale for why this study considered the two as one entity in categorical variables. However, some studies suggest that antiepileptic drugs interact with folic acid and other vitamin B types [55]. Vitamin B varieties in general are known to be neurotrophic factors hence play important role in growth and maintenance of brain functions.

Birth asphyxia, also known as birth injury, was another significant risk factor for the development of brain atrophy. This risk factor had an odd ratio of 1.0 in this study, but many other studies have found that birth asphyxia causes brain volume loss in children. When the injury is severe, it is commonly recognized in neuro-imaging by periventricular hypodensities or what is known as the white cerebella sign [56]. When there is anoxia due to obstructed or difficult labor, the oxygen supply through the basal ganglia and related areas of the cerebral hemispheres is deprived, resulting in tissue damage [57]. It is the necrotic tissues that will be engulfed by macrophages, resulting in brain loss.

This and other studies have shown that reduction in brain volume increases with the increase in age of individuals. Studies have noted that hydration status of brain is more in young age and decreases continuously as age increases [4]. Therefore, all comparative studies of brain volumes in children may put age as a very important parameter that may results in significant variation of brain statuses of children in varying age groups.

In general, it is critical for pediatricians to investigate the previous occurrence of factors such as space-occupying lesions and intracranial pressure, central nervous system infections, head trauma, particularly when associated with loss of consciousness, convulsions and antiepileptic drugs, and birth injury (birth asphyxia) during pediatric neurological disease evaluation and management, as most of these factors, with the exception of malnutrition and inborn errors, are associated with irreversible neurological disease [58]. As a result, primary prevention of these risk factors is critical in terms of mitigating childhood brain atrophy.

## 5. Conclusion

Pathological reduction of brain volume in childhood exhibits a steady trend with the increase in pediatric age.

The most prominent categorical cause of brain atrophy among children in Northern Tanzania is space occupying lesions and intracranial pressure diseases including hydrocephalus.

The additional drivers of brain volume reduction in children include the central nervous system infection, head-trauma, antiepileptic medicines together with convulsive disorders, and birth injuries in particular hypoxic ischemic encephalopathy.
